# Multimodal prediction of neoadjuvant treatment outcome by serial FDG PET and MRI in women with locally advanced breast cancer

**DOI:** 10.1186/s13058-023-01722-4

**Published:** 2023-11-09

**Authors:** Anum S. Kazerouni, Lanell M. Peterson, Isaac Jenkins, Alena Novakova-Jiresova, Hannah M. Linden, Julie R. Gralow, David M. Hockenbery, David A. Mankoff, Peggy L. Porter, Savannah C. Partridge, Jennifer M. Specht

**Affiliations:** 1 Department of Radiology, University of Washington/Fred Hutchinson Cancer Center, Seattle, WA USA; 2 Division of Hematology and Oncology, University of Washington/Fred Hutchinson Cancer Center, 1144 Eastlake (Mail Stop LG-500), Seattle, WA 98109-1023 USA; 3 Fred Hutchinson Cancer Consortium, Seattle, WA USA; 4 Charles University and Thomayer University Hospital, Prague, Czech Republic; 5 American Society of Clinical Oncology, Alexandria, VA USA; 6 Department of Radiology, University of Pennsylvania, Philadelphia, PA USA

**Keywords:** Diffusion weighted MRI, Dynamic contrast enhanced MRI, Dynamic ^18^F-FDG PET, Chemotherapy response, Recurrence

## Abstract

**Purpose:**

To investigate combined MRI and ^18^F-FDG PET for assessing breast tumor metabolism/perfusion mismatch and predicting pathological response and recurrence-free survival (RFS) in women treated for breast cancer.

**Methods:**

Patients undergoing neoadjuvant chemotherapy (NAC) for locally-advanced breast cancer were imaged at three timepoints (pre, mid, and post-NAC), prior to surgery. Imaging included diffusion-weighted and dynamic contrast-enhanced (DCE-) MRI and quantitative ^18^F-FDG PET. Tumor imaging measures included apparent diffusion coefficient, peak percent enhancement (PE), peak signal enhancement ratio (SER), functional tumor volume, and washout volume on MRI and standardized uptake value (SUVmax), glucose delivery (K_1_) and FDG metabolic rate (MRFDG) on PET, with percentage changes from baseline calculated at mid- and post-NAC. Associations of imaging measures with pathological response (residual cancer burden [RCB] 0/I vs. II/III) and RFS were evaluated.

**Results:**

Thirty-five patients with stage II/III invasive breast cancer were enrolled in the prospective study (median age: 43, range: 31–66 years, RCB 0/I: N = 11/35, 31%). Baseline imaging metrics were not significantly associated with pathologic response or RFS (*p* > 0.05). Greater mid-treatment decreases in peak PE, along with greater post-treatment decreases in several DCE-MRI and ^18^F-FDG PET measures were associated with RCB 0/I after NAC (*p* < 0.05). Additionally, greater mid- and post-treatment decreases in DCE-MRI (peak SER, washout volume) and ^18^F-FDG PET (K_1_) were predictive of prolonged RFS. Mid-treatment decreases in metabolism/perfusion ratios (MRFDG/peak PE, MRFDG/peak SER) were associated with improved RFS.

**Conclusion:**

Mid-treatment changes in both PET and MRI measures were predictive of RCB status and RFS following NAC. Specifically, our results indicate a complementary relationship between DCE-MRI and ^18^F-FDG PET metrics and potential value of metabolism/perfusion mismatch as a marker of patient outcome.

**Supplementary Information:**

The online version contains supplementary material available at 10.1186/s13058-023-01722-4.

## Introduction

Neoadjuvant chemotherapy (NAC) is a common strategy for treatment of locally advanced breast cancer to reduce local tumor burden prior to surgery and treat distant micro-metastases. As the number of treatment options has grown with time, the selection of systemic therapies has become increasingly sophisticated, guided by individual patient characteristics and pathological assays [1]. However, response to NAC is variable, with only ~ 35% of patients achieving pathological complete response (pCR), a marker associated with improved event-free and recurrence-free survival [2]. Thus, there is a critical need for early predictors of NAC response to help guide therapy selection for optimal patient outcomes.

Clinical imaging modalities, such as magnetic resonance imaging (MRI) and positron emission tomography (PET), can be used to quantitatively measure physiological tissue properties in vivo*,* allowing for dynamic assessments across a tumor volume [3]. [^18^F]fluorodeoxyglucose (^18^F-FDG) PET, dynamic contrast-enhanced (DCE-) MRI, and diffusion-weighted (DW-) MRI each provide unique measures of the tumor microenvironment that are sensitive to NAC response [4]. ^18^F-FDG PET measures tumor metabolic activity and, through dynamic imaging, can be used to quantify glucose delivery and metabolism [5]. DW-MRI is sensitive to the mobility of water within tissue, quantified as the apparent diffusion coefficient (ADC), a marker reflective of tumor cellularity [6]. DCE-MRI measures signal intensity change over time after intravenous contrast administration, lending insight into lesion vascularity and functional tumor volume (FTV) [7]. Measures derived from each of these modalities have shown promise in the early prediction of NAC response in breast cancer [5, 7–9].

Prior studies have explored the relationship between measures of vascularity and metabolic activity in locally advanced breast cancer [5, 10–14]. Mismatch in the levels of vascular perfusion and metabolic activity may be due to poor vascular delivery and/or elevated metabolism within a tumor, and potentially indicate hypoxic microenvironments associated with therapeutic resistance and were noted to occur more frequently in triple negative breast cancers [13]. Pretherapy mismatch between tumor metabolism and perfusion, measured using ^18^F-FDG and [^15^O]-water PET, was found to be predictive of poor response to NAC [12]. Furthermore, good correlation between dynamic contrast-enhanced (DCE)-MRI kinetics and blood flow measures by ^15^O-water PET in breast tumors [11] suggests DCE-MRI, commonly used for clinical management, could provide a more convenient and translatable method for perfusion assessment [10, 15].

The goal of this prospective study was to evaluate the association of quantitative imaging measures of the tumor microenvironment derived from dynamic ^18^F-FDG PET, DW-MRI and DCE-MRI, including metabolism/perfusion ratios, with NAC response and recurrence-free survival (RFS).

## Methods

### Study population

This prospective study was approved by our Institutional Review Board and compliant with the Health Insurance Portability and Accountability Act (HIPAA). Patients with stage II/III breast cancer who were scheduled to undergo NAC were enrolled in this study (NCT01931709, registration date August 28, 2013) from May 2012 to July 2015. Patients with distant disease beyond regional lymph nodes were excluded. Standard clinical pathological assessment included immunohistochemistry analysis of diagnostic core-needle biopsy specimens for determination of breast cancer subtype based on estrogen receptor (ER), progesterone receptor (PR), and HER2 status (by immunohistochemistry and/or fluorescence in situ hybridization), and Ki-67 proliferation index [16]. Patients were imaged with MRI and ^18^F-FDG PET prior to initiation of treatment, during the NAC (2–12 weeks), and after the completion of NAC.

### Magnetic resonance imaging (MRI)

Patients were imaged on a 3 T Philips Achieva Tx MRI scanner (Philips Healthcare, Best, The Netherlands) equipped with a dedicated 16-channel bilateral breast coil (Mammo-Trak, Philips Healthcare, Best, The Netherlands). Multiparametric breast MRI exams were obtained in the axial orientation and included DW-, and *T*_*1*_-weighted DCE-MRI sequences. DW-MRI was acquired with a single-shot echo-planar imaging sequence with fat suppression, with *b* values of 0, 100, and 800 s/mm^2^. DCE-MRI was acquired with a fat suppressed 3D fast gradient echo (eTHRIVE) sequence, with *T*_*1*_-weighted images were collected before and after administration of gadolinium-based contrast agent (ProHance, bracco Diagnostics, Milan, Italy) at 0.1 mmol/kg body weight. Post-contrast sequences were acquired with *k*-space centered at 2, 5, and 8 min after contrast injection. Additional details regarding image acquisition are provided in the Additional file 1.

### Positron emission tomography (PET)

^18^F-FDG radiotracer was purchased commercially (Cardinal Health, Seattle, WA) or produced in house. PET imaging was performed on an GE Discovery STE PET/CT scanner (GE Medical Systems, Waukesha, WI) as previously described for dynamic imaging [12, 17] with a low dose CT for attenuation correction and positioning. Dynamic imaging was performed over the chest and breast for 60 min after the start of FDG infusion [7–11 mCi (259–407 MBq)] and was followed by a clinical protocol of 5 fields-of-view static imaging. Summed standardized uptake value (SUV) images from 30 to 60 min post injection were constructed from the dynamic data. Additional details regarding image acquisition are provided in the Additional file 1.

### MR image analysis

As previously described [11, 18], DCE-MRI data was analyzed using custom software written in Java and ImageJ (NIH, public domain, Bethesda, MD), providing voxel-wise measures of percent peak enhancement (PE) and signal enhancement ratio (SER). PE was calculated as (*S*_*1 *_− *S*_*0*_)/*S*_*0*_, where *S*_*0*_ and *S*_*1*_ are the pre-contrast and early (2 min) post-contrast signal intensities, respectively. SER was calculated at each voxel, as (*S*_*1 *_− *S*_*0*_)/ (*S*_*2 *_− *S*_*0*_), where *S*_*2*_ is the delayed phase post-contrast image (8 min).

Tumors were segmented in 3D based on percent enhancement of 50% or greater at 2 min post-contrast. A hotspot analysis was performed to identify peak PE and peak SER for each lesion, where a peak was defined as the highest mean value for a 3 × 3 voxel tumor subregion. Additionally, the functional tumor volume (FTV, cc), i.e., the total volume of voxels with PE ≥ 50%, and washout volume (cc), i.e., the total volume of voxels with SER ≥ 1.1, were calculated for each tumor.

DW-MRI data was fit to the conventional monoexponential decay model to calculate the apparent diffusion coefficient (ADC) at each voxel using custom MATLAB-based software (Mathworks, Natick MA). Post-contrast *T*_*1*_-weighted images were used to localize lesions, prior to segmentation of tumor regions-of-interest (ROIs) on the *b* = 800 mm^2^/s image. Lesion ROIs were applied to ADC maps and used to calculate mean ADC for each lesion at each time point.

### PET image analysis

To calculate SUVmax, using the 30–60 min summed images constructed from the dynamic data, volumes-of-interest (VOIs) of approximately 1 cc were drawn on lesions identified on the pre-therapy PET scan encompassing the pixels with the most uptake. Using the pixel with the most uptake, SUVmax was calculated as the maximum tissue activity divided by injected dose/body weight.

Dynamic imaging and kinetic modeling were done as previously described [12, 17]. Briefly, the VOIs drawn on the 30–60 min summed images were applied to the dynamic image set. An approximately 1 cc VOI was also drawn over the left ventricle to create the blood input function. A two-tissue compartment model was utilized to calculate the kinetic parameters using PMOD version 3.6 (Zurich, Switzerland). Metabolic flux (*K*_*i*_), was estimated from parameters derived by fitting the input function and the blood-activity curve to the tissue time-activity curve data, and calculated as (*K*_1 _* *k*_*3*_)/(*k*_*2*_ + *k*_*3*_), where *K*_*1*_ represents the transfer of blood into tissue, *k*_*2*_ is the transport back to blood, and *k*_*3*_ represents metabolic trapping of the tracer. Finally, the metabolic rate of FDG (MRFDG) was calculated as Ki* [Glucose].

### Pathological response and survival outcomes

Pathological response was determined after NAC through histopathological evaluation of the surgical breast specimen by a breast pathologist. Residual cancer burden (RCB) was assessed, with patients categorized as RCB class 0, I, II, or III using methods described by Symmans et al. [19]. Pathological complete response (RCB 0) was defined as no residual invasive disease in the breast or lymph nodes. Following STEEP criteria, RFS was defined as the time between initiation of NAC and disease recurrence or death, whichever came first [20].

### Statistical analysis

Metabolism/perfusion ratios were calculated as the ratio of MRFDG or SUVmax to peak SER or peak PE (i.e., MRFDG/peak SER, MRFDG/peak PE, SUVmax/peak SER, SUVmax/peak PE). Percent change from baseline were calculated for MRI and PET parameters at mid-treatment and post-treatment time points. Spearman rank-order correlation coefficients were calculated between baseline MRI and PET measures. The Wilcoxon rank-sum test was used to compare the percentage change in imaging measures for patients with RCB 0/I versus RCB II/III after NAC. Patients who developed metastases during NAC and did not proceed to surgery were grouped with RCB II/III patients. Univariate Cox proportional hazards regression was used to examine the association of percentage change in each imaging parameter with RFS, with the Wald test used to evaluate each parameter’s estimated hazard ratio. To correct for multiple comparisons, the Benjamini–Hochberg procedure was used to correct *p* values from Wilcoxon rank-sum tests and Cox proportional hazards analyses. Kaplan–Meier curves for RFS were computed for patients dichotomized into two groups based on the third quartile (i.e., 75th percentile) of the percentage change for a given image parameter. Log-rank tests were used to compare survival curves. All statistical analyses were performed in R (R Foundation for Statistical Computing, Vienna, Austria). A *p *value < 0.05 was considered significant.

## Results

### Study population

Thirty-five women (median age: 43, range: 31–66 years) with stage II/III invasive breast cancer were enrolled in the study. Clinical and pathological characteristics of the study population are detailed in Table 1. The majority of patients had Luminal B (N = 20, 57%) or triple negative (N = 12, 34%) subtype. Neoadjuvant chemotherapy was per clinician discretion and represented standard of care for the time period. Of those patients with HER2-negative breast cancer (N = 21), 20 of 21 (95%) received an anthracycline and taxane containing regimen (four with additional platinum agent, and another four with additional investigational agent on the I-SPY2 trial) and one (5%) received taxane (no anthracycline). Of the patients with HER2-positive breast cancer (N = 14), all received taxane and HER2-targeted therapy (e.g., trastuzumab alone, trastuzumab and pertuzumab) based regimens; ten (71%) received additional anthracycline and one (7%) received additional platinum agent. Full treatment details are included in Additional file 1: Table S1. Median duration of chemotherapy treatment was 18 (range: 6–25) weeks, with surgery performed a median of 3.4 (range: 0.1–6) weeks after completion of chemotherapy.

**Table 1:** Patient clinical characteristics and outcomes

Characteristic	No. of Patients (N = 35)	%
Age at diagnosis, years		
30–39	11	31
40–49	13	37
50–59	4	11
60–69	7	20
Race		
Non-Hispanic Caucasian	32	91
Hispanic Caucasian	1	3
Asian/Pacific Islander	2	6
Tumor histology		
Ductal	32	91
Lobular	2	6
Other	1	3
Clinical stage		
IIA	9	26
IIB	8	23
IIIA	9	26
IIIB	3	9
IIIC	6	17
Immunohistochemistry subtype		
Luminal A	1	3
Luminal B	20	57
HER2	2	6
Triple negative	12	34
Residual cancer burden (RCB)		
RCB 0	8	23
RCB I	3	8
RCB II	16	46
RCB III	7	20
Developed metastases prior to surgery	1	3
Long-term outcome		
Recurrences	7	20
Deaths	4	12
Recurrence-free cohort follow up time (years), median (range)	8.1 (1.1–9.8)	

*HR* hormone receptor

Of the 35 study patients, 31% (HER2 N = 1, Luminal B N = 5, triple negative N = 5) achieved RCB 0/I after NAC, 23 were RCB II/III, and one developed metastases and did not undergo surgery (included in RCB II/III group for subsequent analyses). Seven (20%) patients recurred, with a median follow-up time of 8.1 (range: 1.1–9.8) years since treatment initiation. All recurrences were distant metastases, 5 of which were pathologically confirmed metastatic breast cancer (2 assumed but not biopsied) and 4 of which ultimately resulted in death of the patient.

### Correlation between MRI and PET measures

**Fig. 1:**
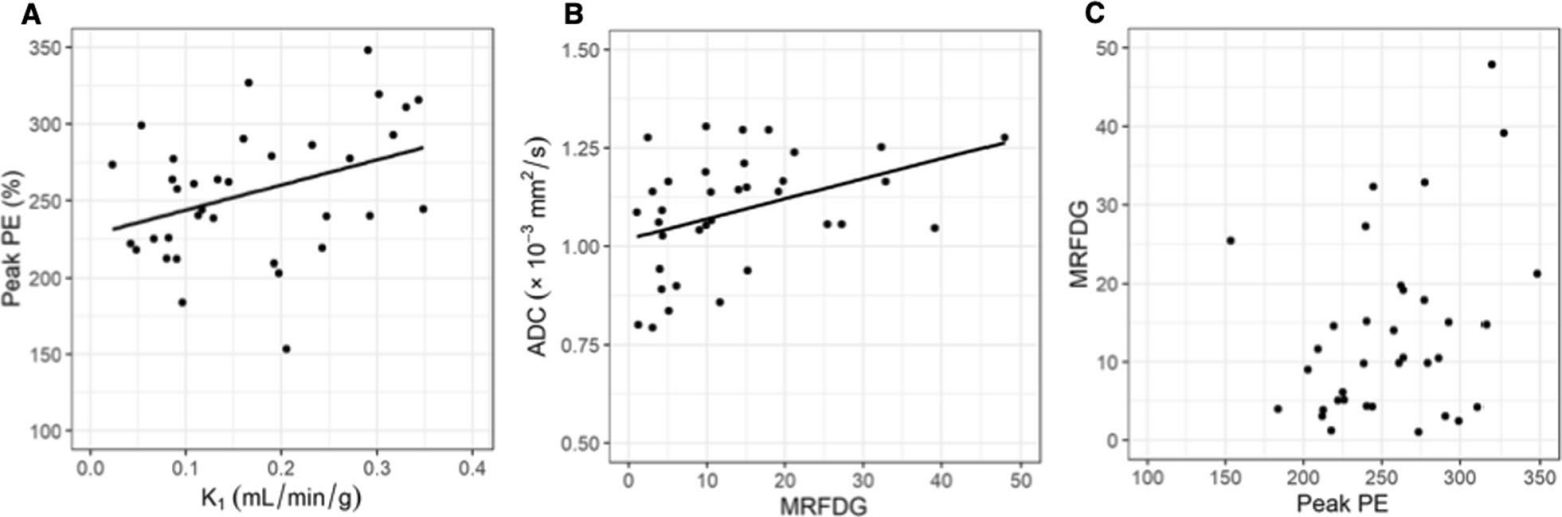
Associations between MRI and PET measures at baseline. **A** K_1_ and peak PE, measures of vascularity and blood flow, showed modest positive correlation (ρ = 0.35, *p* = 0.04). **B** ADC, a measure of cellularity, and MRFDG, a measure of metabolic activity, also showed a modest positive correlation (ρ = 0.38, *p* = 0.03). **C** No significant correlations (*p* > 0.05) were observed between measures of vascularity (peak PE) and metabolic rate (MRFDG)

Vascularity measures by each modality were modestly correlated, including PET K_1_ with DCE-MRI peak PE (ρ = 0.35, *p* = 0.04, Fig. 1A) and peak SER (ρ = 0.31, *p* = 0.07, Additional file 1: Table S2). Additionally, PET measures of metabolic activity (MRFDG and SUVmax) were positively correlated with ADC on DW-MRI, reflecting cellularity (ρ = 0.38, *p* = 0.03, Fig. 1B, and ρ = 0.36, *p* = 0.03, Additional file 1: Figure S1B, respectively). No significant correlations were observed between metabolism and vascularity measures, including MRFDG and peak PE (Fig. 1C) and MRFDG and peak SER (Additional file 1: Figure S1C).

### Association between serial imaging measures and NAC response

**Fig. 2:**
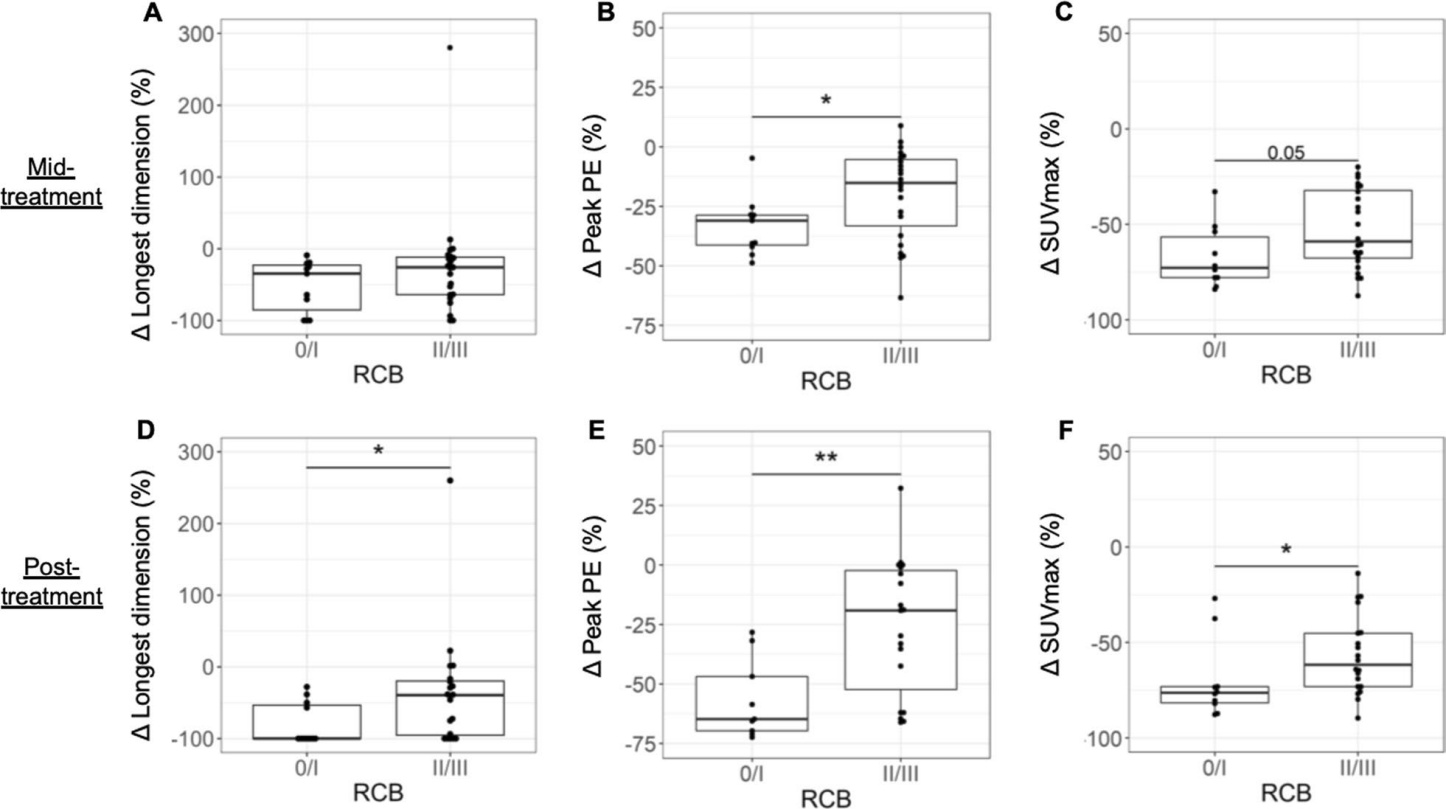
Percentage change from baseline in quantitative imaging measures at mid- and post-treatment. At mid-treatment, no significant differences were observed in change in tumor longest dimension between pathologic responders and non-responders (**A**), while greater decreases in peak PE (*p* = 0.03, **B**) and SUVmax (*p* = 0.05, **C**) were observed in patients who achieved RCB 0/I versus II/III at surgery. At post-treatment, patients who achieved RCB 0/I exhibited a significantly greater decrease in all three imaging metrics (**D**–**F**) compared to patients with RCB II/III disease. *p < 0.05; **p < 0.01

**Fig. 3:**
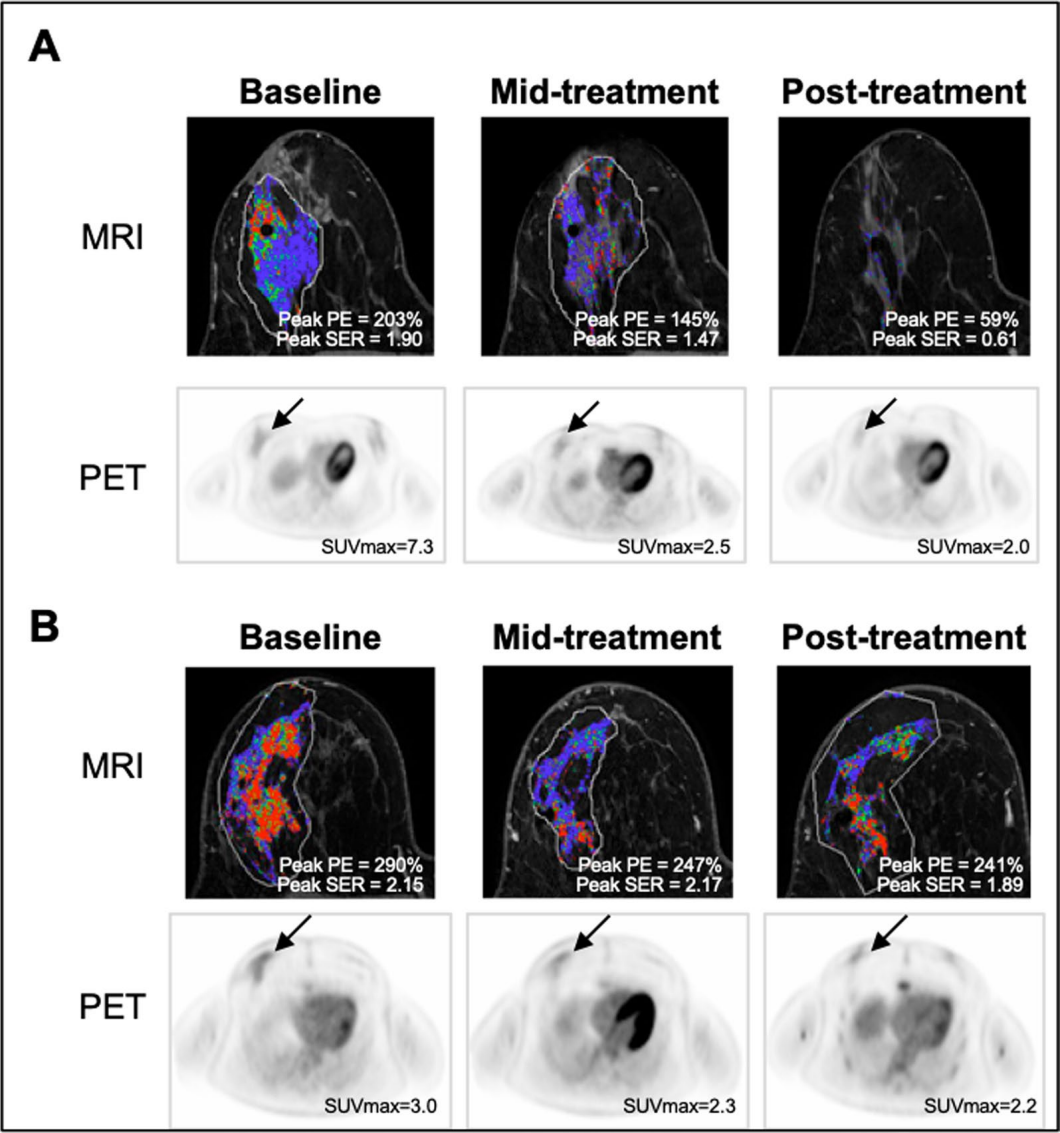
Shown are serial MRI and PET data for example patients: **A** a stage IIIC triple-negative (ER−, PR−, HER2−, Ki67 83%) intraductal carcinoma who achieved RCB 0 after neoadjuvant treatment; **B** a stage IIIA luminal B (ER+, PR− HER2−, Ki67 30%) intraductal carcinoma who had residual disease (RCB II) after neoadjuvant treatment

At baseline, SUVmax was the only imaging measure significantly associated with pathologic response, with RCB 0/I patients demonstrating higher SUVmax than II/III patients (*p* = 0.04, Additional file 1: Table S3). At mid-treatment, percentage change in tumor size (longest dimension on MRI) was not significantly different between RCB 0/I versus II/III patients (Fig. 2A). However, RCB 0/I patients exhibited greater decrease in peak PE compared to RCB II/III patients (− 31.1% vs. − 15.1%, *p* = 0.03, Fig. 2B), as well as a trend of greater decrease in SUVmax (*p* = 0.05, Fig. 2C). At post-treatment, RCB 0/I patients exhibited a significantly greater decrease in K_1_, SUVmax, peak PE, peak SER, and longest dimension (Table 2, Fig. 2D–F, *p* < 0.05 for all). No measures remained significant after adjusting for multiple comparisons. Figure 3 shows serial MRI and PET data for representative study subjects.

**Table 2:** Association of percentage change in quantitative imaging measures with pathologic response to NAC

Percentage change from baseline	Mid-treatment			Post-treatment		
	RCB 0/I N = 11^a^	RCB II/III N = 24^b^	*p*	RCB 0/I N = 11^a^	RCB II/III N = 20^c^	*p*
^18^F-FDG-PET						
K_1_	− 79.9 (14.6)	− 59.1 (46.2)	0.12	− 92 (12.6)	− 75.1 (26.7)	**0.01**
MRFDG	− 86.5 (19.1)	− 80.5 (40.7)	0.40	− 94.1 (13.2)	− 85.8 (23.3)	0.11
SUVmax (30–60 min)	− 72.8 (21.2)	− 59.0 (35.4)	0.05	− 76.3 (8.5)	− 64.1 (28.1)	**0.04**
DW- and DCE-MRI						
ADC	22.2 (20.4)	27.6 (40.0)	0.72	47.5 (34.1)	30.0 (52.9)	0.55
Peak PE	− 31.1 (12.6)	− 15.1 (28.0)	**0.03**	− 65.6 (19)	− 31.4 (55.9)	**0.02**
Peak SER	− 25.4 (21.9)	− 16.9 (27.1)	0.13	− 53.6 (8.6)	− 33.6 (24.5)	**0.01**
FTV	− 81.6 (16.4)	− 61.8 (32.8)	0.11	− 92.3 (7.8)	− 86.5 (27.9)	0.16
Washout volume	− 88.4 (8.5)	− 76.5 (21.4)	0.09	− 95.3 (5.0)	− 93.9 (17.3)	0.18
Longest dimension	− 34.6 (62.4)	− 25.9 (52.2)	0.16	− 100 (46.6)	− 39.4 (75.6)	**0.03**
PET/MRI ratio						
MRFDG/Peak PE	− 80.1 (27.6)	− 72.8 (42.6)	0.50	− 88.1 (41.7)	− 79.6 (54)	0.46
MRFDG/Peak SER	− 81.2 (25.5)	− 75.1 (40.7)	0.48	− 86.7 (27.2)	− 80.7 (45.4)	0.41
MRFDG/K_1_	− 34.2 (87.2)	− 56.0 (77.1)	0.59	− 49.3 (95.0)	− 33.0 (83.3)	0.84
SUVmax/Peak PE	− 57.7 (25.0)	− 44.0 (39.7)	0.25	− 32.6 (72.0)	− 33.6 (50.9)	0.62
SUVmax/Peak SER	− 61.9 (26.1)	− 48.3 (40.7)	0.10	− 49.4 (31.8)	− 37.1 (43.0)	0.76

^a^N = 10 for ^18^F-FDG-PET and PET/MRI ratios

^b^N = 23 for DCE-MRI and PET/MRI ratios, N = 22 for DW-MRI

^c^N = 19 for ^18^F-FDG-PET, N = 18 for PET/MRI ratio

### Association between serial imaging measures and RFS

**Fig. 4:**
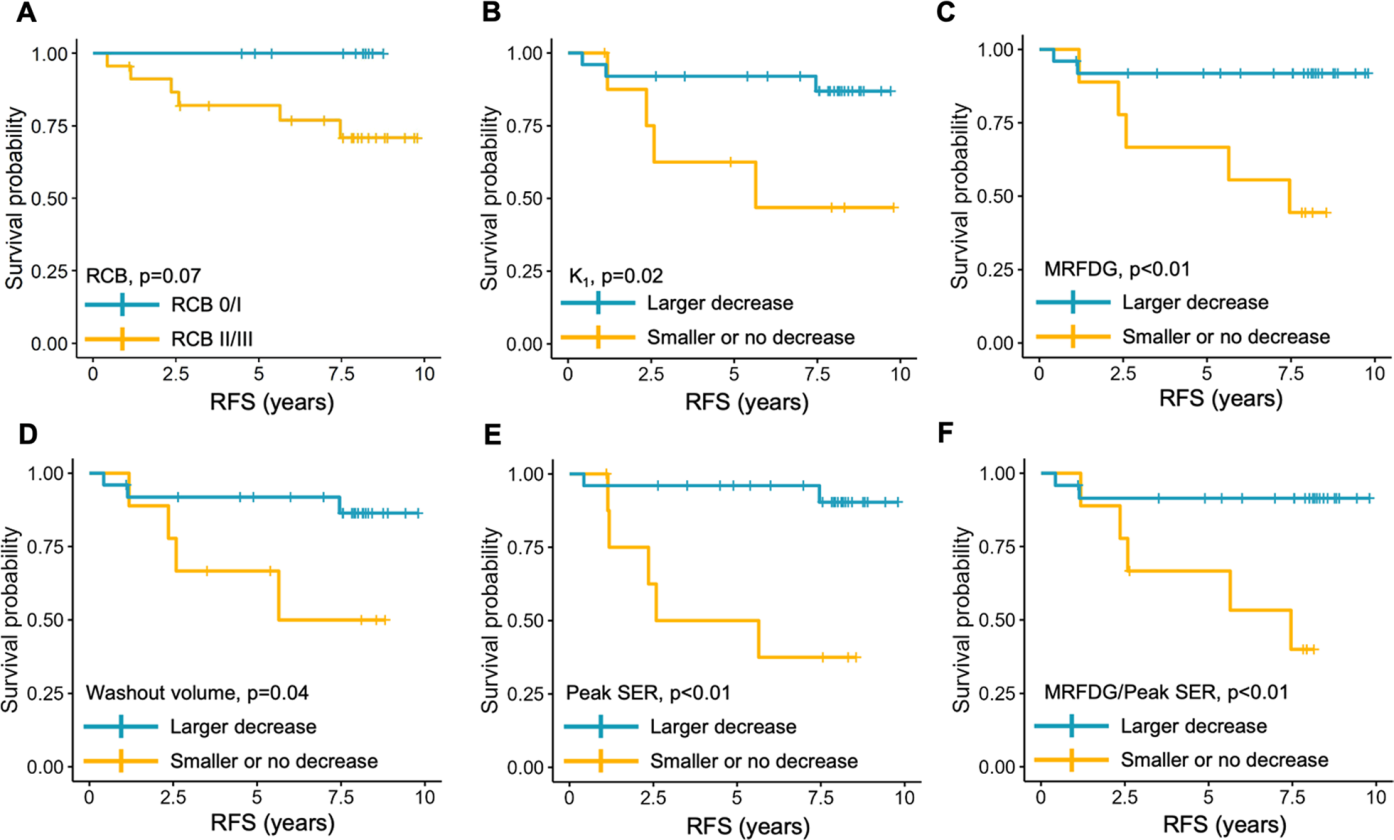
Kaplan–Meier analysis for recurrence-free survival (RFS). Shown are Kaplan–Meier curves stratified by **A** RCB status and mid-treatment percentage change from baseline in **B** K_1_, **C** MRFDG, **D** washout volume, **E** peak SER, and **F** MRFDG/ peak SER. For **B**–**F**, patients were dichotomized at the third quartile for each imaging metric. Patients achieving RCB 0/I tended to exhibit longer RFS versus those with RCB II/III, but the difference did not reach significance (*p* = 0.07). Patients with larger decreases (blue) in K_1_, MRFDG, washout volume, peak SER and MRFDG/peak SER ratio showed longer RFS (*p* < 0.05) compared to those with smaller decreases. Kaplan–Meier curves were compared using the log-rank test

Of the seven patients with disease recurrence, primary tumors in N = 3 were triple-negative and N = 4 were HR+/HER2− subtype; all either had residual disease at surgery (RCB II/III, N = 6) or developed metastases prior to surgery (N = 1). RCB status (0/I vs. II/III) was not significantly associated with RFS by Cox proportional hazards analysis (*p* = 1.00), although patients with RCB 0/I tended to exhibit longer RFS (Fig. 4A, *p* = 0.07). No baseline imaging measures were associated with RFS (*p* > 0.05).

Mid-treatment percentage change in K_1_, MRFDG, washout volume and peak SER each showed significant association with RFS (*p* < 0.05, Table 3), with each 5% increase resulting in 1.03–1.38 fold greater recurrence risk. Additionally, mid-treatment changes in metabolism/perfusion ratios of MRFDG/peak PE and MRFDG/peak SER showed significant associations with RFS (HR = 1.04, *p* = 0.01 for each), with greater decreases associated with reduced risk of recurrence. Kaplan–Meier analysis further confirmed that patients with larger decreases (> 3rd quartile) in K_1,_ MRFDG, washout volume, peak SER, and MRFDG/peak SER at mid-treatment showed reduced risk of recurrence (Fig. 4B–F , p < 0.05) compared to those with smaller decreases. Adjusting for multiple comparisons, mid-treatment percentage change in K_1_, MRFDG, and washout volume remained significantly predictive of RFS (*p* = 0.04). After treatment completion (post-treatment), percentage change in K_1_, peak PE, peak SER, FTV, and washout volume also showed significant association with RFS (*p* < 0.05, Table 3). No post-treatment measures remained significant after adjusting for multiple comparisons.

**Table 3:** Univariate cox proportional hazard analyses for association with RFS

% change from baseline	Mid-treatment				Post-treatment			
	N	Number recurred	HR^a^ (95% CI)	*p*	N	Number recurred	HR^a^ (95% CI)	*p*
K_1_	34	7	1.03 (1.01–1.05)	**< 0.01**^b^	29	6	1.07 (1.02–1.12)	**0.01**
MRFDG	34	7	1.04 (1.01–1.08)	**0.01**^b^	29	6	1.13 (0.98–1.3)	0.09
SUVmax (30–60 min)	34	7	1.19 (0.98–1.45)	0.08	29	6	1.19 (0.98–1.44)	0.07
ADC	33	7	0.94 (0.81–1.08)	0.39	31	6	0.93 (0.81–1.08)	0.35
Peak PE	34	7	1.21 (0.95–1.54)	0.13	31	6	1.19 (1.01–1.39)	**0.03**
Peak SER	34	7	1.38 (1.03–1.84)	**0.03**	31	6	1.26 (1.03–1.55)	**0.03**
FTV	34	7	1.06 (0.94–1.2)	0.33	31	6	1.08 (1.02–1.14)	**0.01**
Washout volume	34	7	1.19 (1.03–1.36)	**0.01**^b^	31	6	1.09 (1.02–1.17)	**0.01**
Longest dimension	34	7	0.99 (0.94–1.06)	0.85	31	6	1.00 (0.96–1.05)	0.72
MRFDG/Peak PE	33	7	1.04 (1.01–1.07)	**0.01**^b^	28	6	1.00 (0.92–1.08)	0.93
MRFDG/Peak SER	33	7	1.04 (1.01–1.07)	**0.01**^b^	28	6	1.03 (0.92–1.15)	0.66
MRFDG/K_1_	34	7	0.98 (0.91–1.05)	0.51	29	6	1.00 (0.95–1.04)	0.87
SUVmax/peak PE	33	7	1.06 (0.93–1.21)	0.38	28	6	1.00 (0.95–1.06)	0.97
SUVmax/peak SER	33	7	1.05 (0.91–1.22)	0.47	28	6	1.03 (0.93–1.14)	0.60

*SUVmax* maximum standardized uptake value, *ADC* apparent diffusion coefficient, *PE* percent enhancement, *SER* signal enhancement ratio, *FTV* functional tumor volume, *RCB* residual cancer burden

^a^1 unit = 5% change

^b^Significant after adjustment for multiple comparisons

## Discussion

Results of our study demonstrated that treatment-induced changes in DCE-MRI and ^18^F-FDG PET measures may predict pathological response and potentially serve as biomarkers of long-term outcome in breast cancer patients undergoing NAC. Our preliminary findings showed mid-treatment decrease in peak PE to be associated with RCB status after NAC, suggesting it may provide an early marker of therapeutic response. Additionally, mid-treatment changes in K_1_, MRFDG, and washout volume were predictive of RFS. Further, when combining PET and MRI measures we observed greater mid-treatment decreases in metabolic/perfusion ratios (MRFDG/peak PE and MRFDG/peak SER) to be associated with longer recurrence-free survival. These findings contribute new insights on the relative value of dynamic PET and MRI markers for assessing therapeutic response in breast cancer.

While current clinical evaluation of response on imaging uses the RECIST criteria [21], based on changes in tumor size, our study shows that these functional imaging measures provide value over established markers of response. Consistent with other studies [5, 10, 22], functional imaging measures from DCE-MRI and ^18^F-FDG PET provided an earlier indication of therapeutic response than changes in tumor size, as longest dimension measures only demonstrated significant differences between RCB 0/I and II/III patients at the later post-treatment timepoint. In our patient cohort, neither RCB nor morphological changes (mid- or post-treatment change in longest dimension) were predictive of long-term outcome. Patients with greater decrease in functional imaging measures at mid-treatment were associated with reduced risk of recurrence, concordant with prior work by our group and others [7, 23]. While we observed longitudinal increases in ADC and FTV over the course of treatment, no significant differences were observed between RCB 0/I vs II/III patients nor significant associations with RFS. This contrasts with findings from larger studies [7, 9] and may be attributable to our small cohort size.

Our study showed vascular measures derived from PET and MRI were generally correlated, confirming previous findings [10]. Further, our results demonstrated no correlation of MRFDG with peak SER or peak PE, which agrees with our prior work establishing a lack of correlation between blood flow and metabolic measures and suggests these measures may provide complementary information [10, 11]. Mismatch between metabolism and perfusion, specifically high metabolism relative to blood flow, may represent increased glucose metabolism and/or poor vascular delivery to the tumor, and potentially contribute to the development of hypoxia and associated chemotherapy resistance [24]. In contrast to the prior PET studies based on ^15^O-water PET perfusion measures [12], we did not find pretherapy metabolism/perfusion mismatch quantified by MRFDG/peak SER and MRFDG/peak PE to be predictive of treatment response. However, mid-treatment changes in these ratios were predictive of outcome, with greater decreases in each associated with longer RFS. Mid-treatment changes in SUVmax/peak SER and SUVmax/peak PE ratios did not show an association with RFS, suggesting that dynamic ^18^F-FDG PET imaging may provide more specific insight into glucose metabolism than standard PET imaging (SUVmax). Interestingly, we observed a modest positive correlation between ADC and MRFDG, suggesting an inverse correlation between cellular density and metabolic activity. While this finding was counterintuitive, other studies investigating the relationship between SUVmax and ADC have shown mixed results [25, 26] and suggest more factors may contribute to ADC than cell density or that some tumor cells may not be metabolically active.

There are some limitations to this study and opportunities for future work. The size of our patient cohort (N = 35) with only seven recurrences limits the statistical power of our findings and precluded multivariate analyses. Differences in patient positioning (supine vs. prone) between the PET and MRI examinations limited direct spatial correlation of the measurements. Furthermore, our image analysis approach to calculate the median values over the entire tumor ROI does not capture potentially useful information on intratumoral heterogeneity. More sophisticated analysis approaches characterizing tumor texture and heterogeneity features [27, 28] could facilitate more comprehensive lesion characterization and measurement of NAC response. Finally, while a research dynamic PET acquisition was performed, for practical reasons DCE-MRI was obtained using the clinical breast MRI protocol, which provided limited temporal sampling and did not allow for more formal pharmacokinetic analysis.

The development of this prospective clinical trial was motivated by prior work [10, 12, 17] to explore the value of metabolism/perfusion mismatch for the prediction of pathological response using perfusion measures derived from more clinically-accessible DCE-MRI. Although our study cohort is modest in size, this unique multimodal data and associated preliminary findings indicating metabolism/perfusion ratios to be associated with RFS may support further investigation in larger cohorts, particularly in the setting of hormone receptor positive breast cancer where pathologic complete response to neoadjuvant therapy is a less robust predictor of event-free survival [29]. Furthermore, our study findings motivate the exploration of emerging hybrid PET/MRI technology, which enables simultaneous PET and MRI acquisition in a single examination, improving the feasibility of such multimodal imaging studies [30]. Additionally, the co-registered data from PET/MRI acquisition may facilitate spatially explicit analyses that could lend novel insights into the spatiotemporal heterogeneity of tumors and response.

## Conclusion

In conclusion, we demonstrate that mid-treatment alterations in tumor microenvironment measures derived from MRI and ^18^F-FDG PET are potential indicators of NAC response and long-term patient outcome, performing better than conventional measures of tumor size. The two modalities offer complementary measures of metabolism and perfusion, and greater reductions in metabolism/perfusion mismatch were associated with improved RFS. These non-invasive imaging-based markers could help guide treatment decisions and facilitate more personalized therapies for optimal patient outcome.

### Supplementary Information

**Additional file 1:** Supplemental Methods.

## Data Availability

The data that support the findings of this study are available on request from the corresponding author, J.M.S.
