# Supplementary material for: Multimodal prediction of neoadjuvant treatment outcome by serial FDG PET and MRI in women with locally advanced breast cancer
Source: Breast Cancer Res. 2023 Nov 9;25:138. doi: 10.1186/s13058-023-01722-4 (PMC10636950; doi:10.1186/s13058-023-01722-4)
Supplement: Supplementary file 1 — Additional file 1: Supplemental Methods. [file 13058_2023_1722_MOESM1_ESM.docx]

**Supplemental Methods**

Magnetic resonance imaging (MRI)

DW-MRI was acquired with a single-shot echo-planar imaging sequence with fat suppression, with the following parameters: TR = 5000-6000 ms; TE = 59-62 ms; spatial resolution = 1.5 × 1.5 to 1.9 × 1.9 mm; slice thickness = 4-5 mm. Diffusion gradients were applied in six directions with *b* values of 0, 100, and 800 s/mm^2^. DCE-MRI was acquired with a fat suppressed 3D fast gradient echo (eTHRIVE) sequence, with the following parameters: TR = ~6 ms; TE = ~3 ms; flip angle = 10º; spatial resolution = 0.5 × 0.5 to 0.6 × 0.6 mm; slice thickness = 1.3 mm. *T_1_*-weighted images were collected before and after administration of gadolinium-based contrast agent (ProHance, bracco Diagnostics, Milan, Italy) at 0.1 mmol/kg body weight. Post-contrast sequences were acquired with *k*-space centered at 2, 5, and 8 minutes after contrast injection.

Positron emission tomography (PET)

Following a low dose CT for attenuation (60mA/2.5mm), PET images were acquired according to standard machine characteristics. The slice thickness was 3.27mm and a reconstruction diameter of 550mm. Array size (pixels) was 128x128 and pixel size of 5.46mm and volume of 2.64cc. 2D Filter Back Projection (FBP) reconstruction used a Hanning, 7mm convolution kernel. A one-minute injection of FDG was followed by dynamic imaging. The dynamic image sequence was as follows: 4x20s, 4x40s, 4x1m, 4x3m, 8x5m, and was followed by a standard clinical 5 field-of-view static torso scan.

**Figure S1**


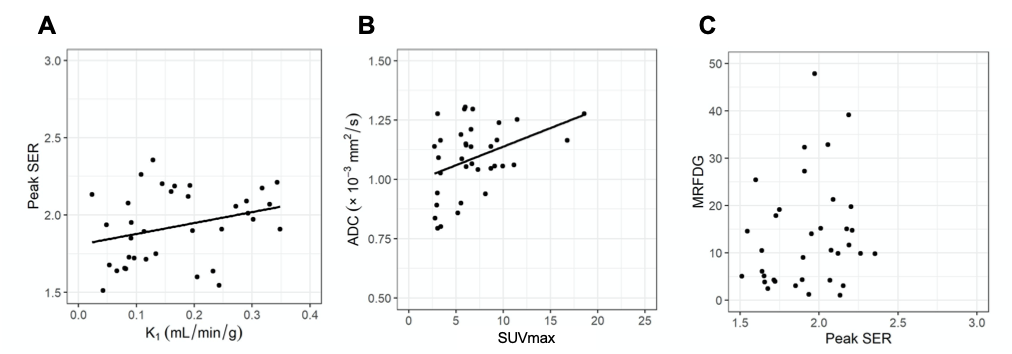


**Figure S1**: Additional associations between MRI and PET measures. A) K_1_ and peak SER, showed modest positive correlation, trending towards significance (𝜌=0.31, p=0.07). B) ADC and SUVmax showed a modest positive correlation (𝜌=0.36, p=0.03). C) No significant correlations (p>0.05) were observed between peak SER and MRFDG.

**Table S1: Baseline (pre-therapy) differences between response groups**

| **Subject** | **Subtype** | **Neoadjuvant treatment** |
| --- | --- | --- |
| 1 | Triple-negative | ACT |
| 2 | Triple-negative | ACT |
| 3 | Triple-negative | ACT |
| 4 | Triple-negative | ACT |
| 5 | Triple-negative | ACT |
| 6 | Triple-negative | ACTP |
| 7 | Triple-negative | ACTP |
| 8 | Triple-negative | ACTP |
| 9 | Triple-negative | ACTI |
| 10 | Triple-negative | ACTI |
| 11 | Triple-negative | ACTI |
| 12 | Triple-negative | CTx |
| 13 | Luminal A | ACT |
| 14 | Luminal B | ACT |
| 15 | Luminal B | ACT |
| 16 | Luminal B | ACT |
| 17 | Luminal B | ACT |
| 18 | Luminal B | ACT |
| 19 | Luminal B | ACTI |
| 20 | Luminal B | ACTP |
| 21 | Luminal B | ACTH |
| 22 | Luminal B | TH |
| 23 | Luminal B | ACTH |
| 24 | Luminal B | ACTH |
| 25 | Luminal B | ACTH |
| 26 | Luminal B | ACTH |
| 27 | Luminal B | ACTH |
| 28 | Luminal B | ACTH |
| 29 | Luminal B | ACTH |
| 30 | Luminal B | ACTH |
| 31 | Luminal B | TPH |
| 32 | Luminal B | TH |
| 33 | Luminal B | TH |
| 34 | HER2+ | ACTH |
| 35 | HER2+ | ACTH |

A: Adriamycin; C: Cytoxan; T: Taxol; P: Platinum-based treatment; Tx: Taxotere; H: HER2-targeted; I: ISPY-2 Investigational agent;

**Table S2:** Correlation between MRI and PET measures.

|  | 𝜌 | *p** |
| --- | --- | --- |
| K_1_ and peak PE | 0.35 | 0.04 |
| K_1_ and peak SER | 0.31 | 0.07 |
| ADC and MRFDG | 0.38 | 0.03 |
| ADC and SUVmax | 0.36 | 0.03 |
| Peak PE and MRFDG | 0.19 | 0.09 |
| Peak SER and MRFDG | 0.29 | 0.26 |

*, Spearman rank-order correlation

**Table S3: Baseline (pre-therapy) differences between response groups**

|  |  | Residual Cancer Burden | | |
| --- | --- | --- | --- | --- |
|  |  | RCB 0/I  N=11 | RCB II/III  N=24 | *p** |
| ^18^F-FDG-PET | |  |  |  |
|  | K_1_ (mL/min/g) | 0.2 (0.1) | 0.1 (0.2) | 0.87 |
|  | MRFDG (μmol/min/100g) | 9.8 (19.3) | 10.5 (10.9) | 0.47 |
|  | SUV max (at 30 min) | 7.3 (4.5) | 5.7 (3.8) | 0.04 |
| DW- and DCE-MRI | |  |  |  |
|  | ADC (×10^-3^ mm^2^/s) | 1.2 (0.2) | 1.1 (0.2) | 0.11 |
|  | Peak PE (%) | 244.5 (60.2) | 259.9 (63.1) | 0.39 |
|  | Peak SER | 1.9 (0.3) | 2.0 (0.4) | 0.19 |
|  | FTV (cc) | 10.9 (25.9) | 12.8 (26.4) | 0.45 |
|  | Washout volume (cc) | 2.5 (3.7) | 5.0 (9.5) | 0.14 |
|  | Longest dimension (mm) | 34 (13.5) | 41 (29.5) | 0.43 |
| PET/MRI Ratio | |  |  |  |
|  | MRFDG / Peak PE | 0.04 (0.1) | 0.04 (0.04) | 0.29 |
|  | MRFDG / Peak SER | 4.8 (11.2) | 5.2 (5.6) | 0.31 |
|  | MRFDG/K_1_ | 92.7 (70.2) | 55.8 (59.2) | 0.16 |

Shown are median (IQR)

*, Wilcoxon rank sum test
